# Molecular phylogeny of coronaviruses and host receptors among domestic and close-contact animals reveals subgenome-level conservation, crossover, and divergence

**DOI:** 10.1186/s12917-022-03217-4

**Published:** 2022-04-01

**Authors:** Kingsley Bentum, Sage Shaddox, Crystal Ware, Gopal Reddy, Woubit Abebe, Raphael Folitse, Pamela Martin, Temesgen Samuel

**Affiliations:** 1 Department of Pathobiology, College of Veterinary Medicine, Patterson Hall, Tuskegee University, Patterson Hall, 1200 W. Montgomery Road, Tuskegee, AL 36088 USA; 2 School of Veterinary Medicine, Kwame Nkrumah University of Science and Technology, University Post Office, Kumasi, Ghana

**Keywords:** Coronavirus, Host receptors, Comparative phylogeny, Furin cleavage, Spike protein

## Abstract

**Background:**

Coronaviruses have the potential to cross species barriers. To learn the molecular intersections among the most common coronaviruses of domestic and close-contact animals, we analyzed representative coronavirus genera infecting mouse, rat, rabbit, dog, cat, cattle, white-tailed deer, swine, ferret, mink, alpaca, Rhinolophus bat, dolphin, whale, chicken, duck and turkey hosts; reference or complete genome sequences were available for most of these coronavirus genera. Protein sequence alignments and phylogenetic trees were built for the spike (S), envelope (E), membrane (M) and nucleocapsid (N) proteins. The host receptors and enzymes aminopeptidase N (APN), angiotensin converting enzyme 2 (ACE2), sialic acid synthase (SAS), transmembrane serine protease 2 (TMPRSS2), dipeptidyl peptidase 4 (DPP4), cathepsin L (and its analogs) and furin were also compared.

**Results:**

Overall, the S, E, M, and N proteins segregated according to their viral genera (α, β, or γ), but the S proteins of alphacoronaviruses lacked conservation of phylogeny. Interestingly, the unique polybasic furin cleavage motif found in severe acute respiratory syndrome coronavirus-2 (SARS-CoV-2) but not in severe acute respiratory syndrome coronavirus (SARS-CoV) or Middle East respiratory syndrome coronavirus (MERS-CoV) exists in several β-coronaviruses and a few α- or γ-coronaviruses. Receptors and enzymes retained host species-dependent relationships with one another. Among the hosts, critical ACE2 residues essential for SARS-CoV-2 spike protein binding were most conserved in white-tailed deer and cattle*.*

**Conclusion:**

The polybasic furin cleavage motif found in several β- and other coronaviruses of animals points to the existence of an intermediate host for SARS-CoV-2, and it also offers a counternarrative to the theory of a laboratory-engineered virus. Generally, the S proteins of coronaviruses show crossovers of phylogenies indicative of recombination events. Additionally, the consistency in the segregation of viral proteins of the MERS-like coronavirus (NC_034440.1) from pipistrelle bat supports its classification as a β-coronavirus. Finally, similarities in host enzymes and receptors did not always explain natural cross-infections. More studies are therefore needed to identify factors that determine the cross-species infectivity of coronaviruses.

## Background

The ongoing pandemic caused by severe acute respiratory syndrome coronavirus-2 (SARS-CoV-2) has led to unprecedented interest in the study of coronaviruses, although the first coronavirus was identified in the 1930s [1]. Given the most recent outbreaks by SARS-CoV-2, severe acute respiratory syndrome coronavirus (SARS-CoV) and Middle East respiratory syndrome coronavirus (MERS-CoV), the potential for zoonoses and reverse zoonoses, in conjunction with viral, host and environmental factors, have elevated the need to identify the origin, transmission, pathogenesis and control of and novel therapeutic and preventive strategies against these viruses.

The first two-thirds of the coronavirus genome encodes proteins needed for replication, and the remaining one-third encodes accessory and structural proteins, which include hemagglutinin esterase (HE) (present in only Group 2 coronaviruses), spike (S), envelope (E), membrane (M) and nucleocapsid (N) [2, 3]. Previously grouped based on serology, coronaviruses are now categorized into α (alpha), β (beta), γ (gamma) and δ (delta) groups using genetics. Coronaviruses affect many host species ranging from mammals to birds [3, 4].

Cross-species coronavirus infections have been reported among humans and animals. The SARS-CoV epidemic in 2002, for example, appears to have jumped from bats to infect palm civets, then to raccoon dogs, Chinese ferret badgers and finally into the human population [5]. In domestic animals, the most likely recent interspecies transmission leading to an outbreak may be that of canine respiratory coronavirus discovered in 2003 [6]. In Africa, a recent study by Burimuah et al. also showed that the close association of ruminants can lead to a spillover of coronaviruses from cattle to other small ruminants [7]. A high degree of sequence identity among some canine, bovine and human β-coronaviruses [2, 5, 8] strongly suggests that coronaviruses in close-contact environments could recombine or jump the species barrier to initiate emerging infections with sustained transmissions in new hosts. In the last two decades, SARS-CoV-2 has been the third zoonotic coronavirus to originate from animals and cause a pandemic in humans. Although the intermediate host for SARS-CoV-2 has not yet been identified [9, 10], recent comparative molecular analysis indicates that the pangolin and bats harbor the closest relative viruses [11–14].

With the current public availability of whole-genome sequences and additional tools for the analysis of both proteome and genome data, it is now possible to examine viruses and other microbes in great detail even before experimental validations. In this study, we analyzed the phylogenetic relationships among selected coronaviruses that infect domestic and close-contact animals to sketch the subgenomic relationships among the viruses. The molecular phylogeny of coronavirus proteins (spike, envelope, membrane and nucleocapsid), putative host-cell receptors and key functional domains of host enzymes were compared.

## Results

### Viruses of the same genera may form variable clades at subgenomic levels

**Fig. 1:**
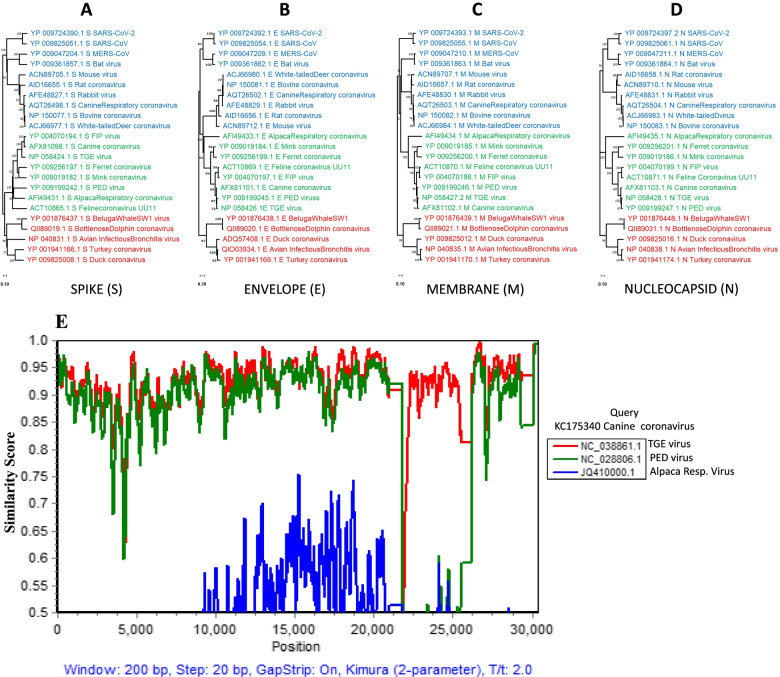
Clustering patterns for representative α, β, or γ coronaviruses in domestic and close-contact animals. Phylogenetic trees built for the **A** spike (S), **B** envelope (E), **C** membrane (M) and **D** nucleocapsid (N) proteins. Viruses are clustered according to their α, β, or γ groupings. *MERS-like coronavirus (PREDICT/PDF-2180, Acc #: NC_034440.1) currently not belonging to a classified coronavirus is seen clustering among β-coronaviruses. **E** SimPlot analysis showing the similarity score between the whole genomes of canine coronavirus, PED virus, TGE virus and alpaca respiratory coronavirus with canine coronavirus (also referred to as canine enteric coronavirus) set as the query sequence

Overall, viral S, E, M, and N proteins clustered together according to the viral genera groups (Fig. 1 A-D), although intragroup discordances were evident. For alpaca respiratory α-coronavirus, the S protein shared the same origin as that of the porcine epidemic diarrhea (PED) virus, but distinct from its clade, the E protein shared a distant origin with β-coronaviruses (Fig. 1B). Among β-coronaviruses, proteins from canine respiratory, bovine, rabbit, and white-tailed deer coronaviruses were closely related. The E, M, and N proteins from transmissible gastroenteritis (TGE) virus, canine coronavirus (also referred to as canine enteric coronavirus) and PED virus showed close phylogeny with each other, while the S protein showed discordance. Feline coronavirus (strain UU11) and feline infectious peritonitis (FIP) virus E, M, and N proteins were closely related (Fig. 1 B-D). However, the phylogeny of the S protein from FIP virus was distant from that of feline coronavirus (strain UU11) (Fig. 1 A). To test for possible past recombination events that may have led to the crossover phylogeny between viruses of unrelated host species, we performed a SimPlot analysis of the entire genomes of canine coronavirus, PED virus, TGE virus and alpaca respiratory coronavirus, holding the canine coronavirus sequence as a query against the other three. The results showed that both TGE- and PED-coronaviruses maintained 70-98% similarity with the canine coronavirus along the genome region (approximately 20,000 bases) proximal to the segment coding for the S protein. However, in the S gene segment, the PED virus showed no similarity to either canine coronavirus or TGE virus. TGE virus regained 90-96% similarity to canine coronavirus after a drop of the curve in the proximal region of the S gene (Fig. 1E). The alpaca respiratory coronavirus shared very limited similarity with the canine coronavirus between the proximal 10,000 and 20,000 bases but was similar to the PED virus between the 23,000 and 25,000 base marks corresponding to the spike gene segment. This suggests that the S gene segments of some coronaviruses may originate from other viruses via recombination, leading to divergent subgenome phylogeny among proteins of the same virus.

### The polybasic furin cleavage motif in SARS-CoV-2 is present in several β-coronaviruses

**Fig. 2:**
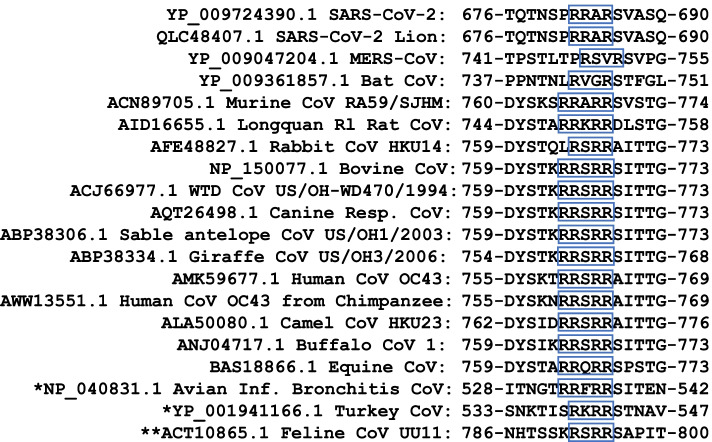
Polybasic furin cleavage motif of SARS-CoV-2 is present in other coronaviruses. The protein sequence of the furin cleavage site within the S protein, with polybasic amino acid residues conserved among some coronaviruses, is shown. The residues constituting the RRXR motif (where R is an arginine residue and X is another amino acid) are marked with rectangles. The exact RRAR configuration of this motif in SARS-CoV-2 is present in the murine coronavirus but absent in others, including the bat virus and MERS-CoV. The RRXR motif is reversed in others such as the rabbit HKU14 coronavirus and turkey coronavirus. Amino acids are listed in single letter code

In SARS-CoV-2, the S protein contains a polybasic furin cleavage site with the motif RRAR (where R and A are arginine and alanine residues, respectively). This motif is present in the murine β-coronavirus but absent in SARS-CoV, while MERS-CoV has an RSVR sequence at approximately the same location. This cleavage site, located at the junction of the S1/S2 subdomains, specifically has an arginine residue at the second position (R**R**AR), which is essential for efficient cleavage of the S protein [15]. We show that this furin cleavage motif RRXR (where X is an alanine residue or another amino acid) is present in several β-coronaviruses and the avian infectious bronchitis virus (a γ-coronavirus) as shown in Fig. 2. Notably, a variation in the sequence is also evident in other S proteins, such as RVGR in a β-coronavirus of bat, RSRR in an α-coronavirus of cat, and RKRR in a γ-coronavirus of turkey. Therefore, this motif or its mutant variants exist in natural coronaviruses belonging to different groups.

### The TMPRSS2 cleavage site on the viral S protein and the catalytic residues of the host TMPRSS2 enzyme are conserved

**Fig. 3:**
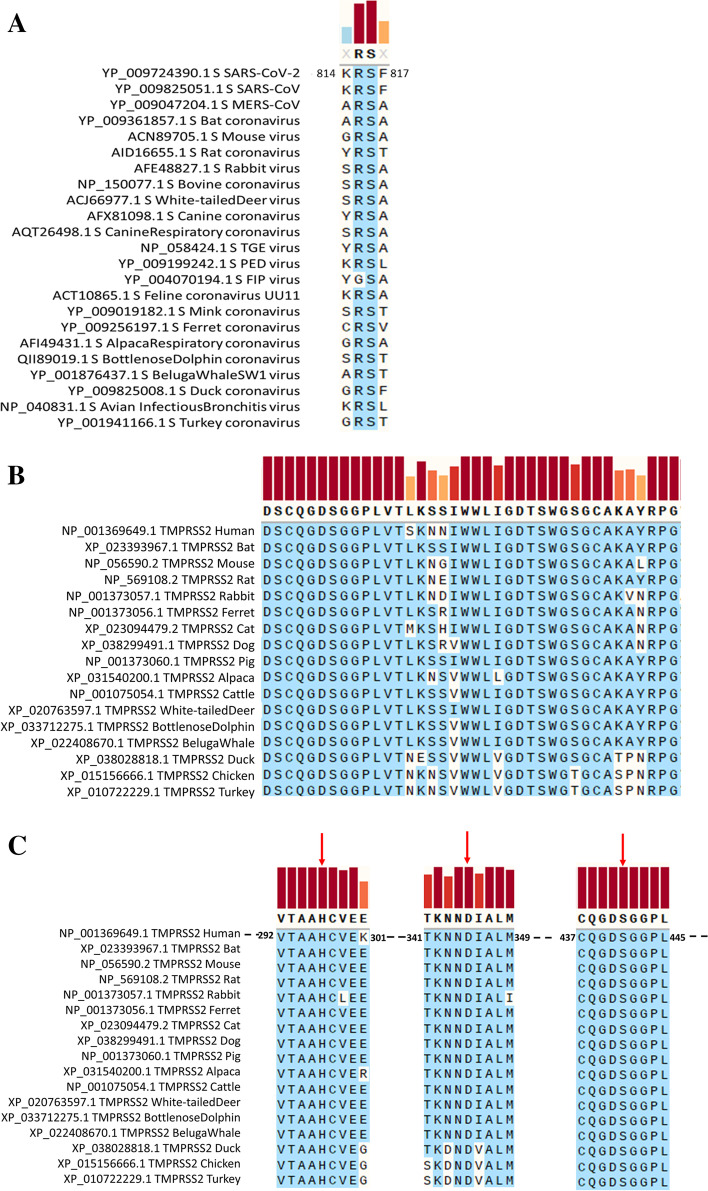
S protein cleavage site and catalytic residues of the TMPRSS2 enzyme are conserved. **A** S protein sequence alignment at the site where human TMPRSS2 cleaves the SARS-CoV-2 spike protein. The TMPRSS2 enzyme cleaves between the arginine (R) and serine (S) residues. Only the FIP virus has a glycine (G) in place of the arginine (R) residue. Aligned protein sequences of the substrate binding site (**B**) and active site (**C**) of the TMPRSS2 enzyme of various host species are shown. Arrows in panel **C** point to the triad of catalytic residues histidine (H) 296, aspartic acid (D) 345 and serine (S) 441 that are essential for binding to the SARS-CoV-2 S protein. All conserved residues are shaded blue, and sequence consensus conservation is shown as colored bars (red, tall bars mean more conserved). The threshold for showing a consensus is set at > 70 for **A** and > 50% for **B** and **C**. The letter X denotes no consensus, and amino acids are listed in single letter codes

The TMPRSS2 cleavage site of the SARS-CoV-2 S protein is well conserved among all viruses studied, although the residues flanking the cleavage site vary widely (Fig. 3A). TMPRSS2 is expressed in tissues of the aerodigestive tract of humans [16], and it is important for initiating SARS-CoV-2 infection by processing the S protein to release fusion peptides for membrane fusion [17]. On the host TMPRSS2 enzyme, we found both the substrate binding site and the triad of catalytic residues (histidine (H) 296, aspartic acid (D) 345 and serine (S) 441) located in the active site to be well conserved among all host species as shown in Fig. 3B and C, respectively.

### Comparison of host ACE2, TMPRSS2, APN SAS, DPP4, cathepsin L and furin proteins

**Fig. 4:**
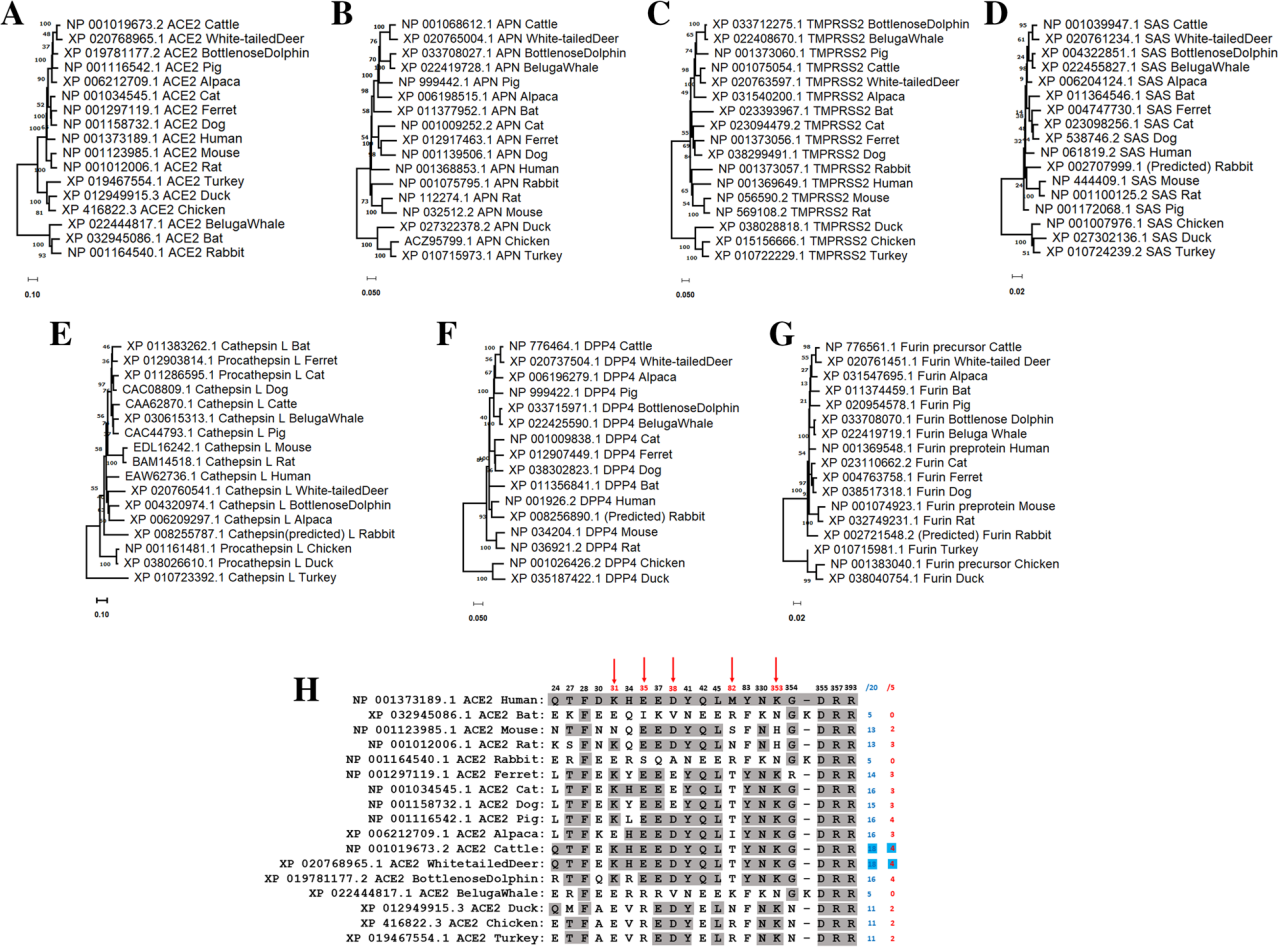
Comparisons of phylogenetic relationships among host receptors and enzymes. Phylogenetic comparison of **A** ACE2, **B** APN, **C** TMPRSS2, **D** SAS, **E** DPP4, **F** cathepsin L/procathepsin L and **G** furin among various host species. Generally, the phylogenetic segregation of host enzymes and receptors followed the species-related pattern except for the ACE2 enzyme, which also showed the highest genetic variation scale of 0.10. **H** Comparison of the key amino acids in human ACE2 projected to interact with the receptor binding domain of the S protein of SARS-CoV-2. Shaded in dark gray and arrowed in red are the positions and the single letter codes of the 20 amino acids and 5 critical residues, respectively, required for successful S protein attachment. The proportion of amino acids that overlap with the twenty required (/20) and the 5 critical (/5) residues are shown on the right in blue and red, respectively. The fractional scores for cattle and white-tailed deer are colored in aqua

Phylogenetic analysis of host enzymes and receptors was performed. TMPRSS2, SAS, APN, DPP4, cathepsin L (and its analogs) and furin showed high similarity among mammals, with distinct separation from those of birds. An overall species-based segregation pattern was observed for the various host enzymes and receptors, except for the ACE2 enzyme, where bat, rabbit and beluga whale ACE2 proteins were distantly related to proteins of other mammals and birds (Fig. 4A-G). Unlike the phylogenies of host species cathepsin and furin, which generally followed species relationship patterns, the phylogeny of DPP4 was rather peculiar in that proteins of cattle and white-tailed deer or beluga whale and bottlenose dolphin were very distantly related. On the other hand, cathepsins of dog and cattle were in the same clade (Fig. 4 E-G). Upon analyzing both the required and critical residues of ACE2 needed for SARS-CoV-2 S protein binding, the cattle and white-tailed deer proteins shared the most similarity scores with humans, followed closely by the dolphin and pig proteins and then by the cat, alpaca and dog proteins (Fig. 4H).

## Discussion

We compared the protein-level phylogenetic relationships among common coronaviruses of domestic and close-contact animals and key infection-associated proteins in the hosts. In this report, we highlight that the polybasic furin cleavage site found in SARS-CoV-2, but not in SARS-CoV or MERS-CoV [15], exists in several β-coronaviruses included in this report, although the configuration varied in some of those. For example, in the murine coronavirus (accession no. ACN89705.1), the exact RRAR motif is observed, but in others, it is either reversed or palindromic (Fig. 2). The polybasic furin cleavage site in the SARS-CoV-2 S protein is considered unique to the novel virus that causes COVID-19 [18]. This has led to speculations about the possibility of a laboratory-engineered virus. However, the presence of the same and similar motifs in other β-coronaviruses and even other coronaviruses eliminates the theory of an artificially engineered virus. Although the current search for the intermediate host of SARS-CoV-2 suggests several potential candidates [19], possible recombination events of viruses in the same or different host species, which can give rise to a hybrid or a variant species, should not be ruled out. RpYN09 bat coronavirus, the most recently found closest relative of SARS-CoV-2, lacks the RRAR motif [14], suggesting that the parent virus containing this motif has yet to be discovered.

Although the identities of all host receptors for coronaviruses are not fully known, ACE2 is documented as a receptor mediating both infection and transmission of SARS-CoV-2 [20, 21]. ACE2 is a key regulator of the angiotensin system and is well expressed in the vascular endothelium, smooth muscle cells of the intestines, kidneys and heart muscle cells [22–24]. During infection, twenty key amino acid residues in the ACE2 enzyme interact with the S protein of SARS-CoV-2 [25]. Five of these residues, lysine (K), glutamic acid (E), aspartic acid (D), methionine (M) and lysine (K), at positions 31, 35, 38, 82 and 353, respectively, are critical for S protein binding [26]. Sequence alignment analysis showed that cattle and white-tailed deer share the most similarity scores to humans for both required and critical amino acid residues. Interestingly, a recent USDA/APHIS study showed that approximately 40% of the wild white-tailed deer population in four states in the USA tested positive for anti-SARS-CoV-2 immunoglobulins (https://www.aphis.usda.gov/animal_health/one_health/downloads/qa-covid-white-tailed-deer-study.pdf). This finding indicates the possibility that SARS-CoV-2 may establish and spread in hosts with high affinity receptors. However, the similarity of a single receptor may not explain or predict cross-species infections. For example, while the bovine-canine cross-species jump of a β-coronavirus is obvious from the phylogeny, the comparison of their ACE2 does not indicate a unique relationship of this receptor between cow and dog, although their cathepsins belong to the same clade. Therefore, ACE2 may not necessarily be a receptor for bovine-canine interspecies viral infection. Similarly, ACE2 of bottlenose dolphin and pig shared more common residues with those of human ACE2 than other hosts. Specifically, the Rhinolophus bat reference sequence has one of the least phylogenetic and key amino acid commonalities with human ACE2 (Fig. 4H). This brings into question the relevance of the ACE2 receptor in this bat species.

The TMPRSS2 cleavage site in SARS-CoV-2 was conserved among all viruses we included (Fig. 3A). In the host species, both the substrate binding and active sites of the enzyme were well conserved. Notably, the triad of catalytic residues histidine (H) 296, aspartic acid (D) 345 and serine (S) 441 [27], which interact with the SARS-CoV-2 S protein, are conserved among all species included in this study (Fig. 3B). The overall phylogenetic segregation pattern of host enzymes and receptors was species-related except for the ACE2 enzyme, where bat, rabbit and whale proteins were distantly related to those of other mammals.

Among the viruses we analyzed, consistent patterns with little variation were observed among γ-coronaviruses. The canine respiratory, rabbit, bovine respiratory, and white-tailed deer coronaviruses also consistently clustered together. However, phylogenetic crossovers were evident among canine coronavirus, TGE virus, PED virus, feline coronavirus, and FIP virus. The S protein of the feline coronavirus included in this report was distant from that of the FIP virus, displaying discordant relationships that indicate past recombination events [28]. An interesting phylogenetic common origin was also noted for the S proteins of PED virus and the distantly related alpaca respiratory coronavirus. These crossovers are most likely from past recombination and mutation events. Coronaviruses are noted for their high frequency of recombination [29], and the patterns of recombination among coronaviruses of domestic animals deserve a closer look for us to recognize the origins of cross-species infections and to design effective disease control strategies.

Finally, we also noted that the MERS-like bat coronavirus (accession number YP_009361857.1; https://www.ncbi.nlm.nih.gov/protein/1189488876) is annotated as unclassified in the National Center for Biotechnology Information (NCBI) database. Considering the phylogenetic clustering of the S, M, E, and N proteins, they most likely belong to the β-coronavirus group.

Limitations of our study include the unknown nature of the complete receptor repertoire for coronaviruses among domestic and close-contact animals. Although we based our comparative phylogeny primarily on the reported or putative human receptor or coronavirus infection-associated proteins, coronaviruses may not use the same receptors in different species. It is possible that a virus crossing to another species will adapt to using different receptors or associated proteins.

## Conclusion

Recombination or a few critical mutations in viral receptor binding domains or receptor protein key residues may contribute to cross-species infections [30]. We highlighted key molecular relationships that exist among common coronaviruses and their host receptors. Notably, the existence of similar polybasic residues within the S proteins of several coronaviruses suggests that the RRXR sequence motif is not unique to SARS-CoV-2. Unlike genome-level phylogenies, subgenomic-level comparisons provide stronger insights into functional ontogeny and the identification of consequential recombination events. However, not all factors that determine species-specific or cross-species infections are currently known. Therefore, additional studies of host determinants for virus attachment and productive infectivity are urgently needed to understand the role different hosts play in coronavirus infections, maintenance, and host adaptations. Such studies will be critical to identify molecules that can be targeted for the prevention and control of diseases caused by these viruses, especially in hosts of economic or public health importance.

## Methods

All protein sequences included in our analysis were collected from the National Center for Biotechnology Information (NCBI) database (https://www.ncbi.nlm.nih.gov/nuccore/), stored in FASTA formats and analyzed. In total, 32 coronaviruses, comprising eight α-coronaviruses, 19 β-coronaviruses and five γ-coronaviruses, were included in our comparative study as shown in Table 1. For each of the viruses, we assembled protein sequence data on the spike (S), membrane (M), envelope (E) and nucleocapsid (N) structural proteins. Furthermore, protein sequence data on key receptors and enzymes reported to be involved in viral infection, namely, angiotensin converting enzyme 2 (ACE2), transmembrane serine protease 2 (TMPRSS2), aminopeptidase N (APN), sialic acid synthase (SAS), dipeptidyl peptidase 4 (DPP4), cathepsin L and furin, of seventeen hosts were also assembled, and their details are shown in Table 2. The FASTA format of all sequences was organized in Text Editor for analyses. Using MEGA X software version 11, sequence alignments and phylogenetic trees were developed. Alignments were generated using the Muscle Tool with neighbor-joining as the cluster method, and the degree of consensus among conserved residues was visualized using Snap Gene software. The SimPlot program [31] was used to graphically depict similarities among selected sequences. Phylogenetic trees were constructed using the neighbor-joining method with a Poisson model of substitution at 1000 bootstrap replications, and all the results were validated with the maximum likelihood method. All other default settings of the bioinformatics programs used were applied.

**Table 1:** Virus sequences included in this study and their respective hosts

Virus Group	Name of Virus	Accession ID	Host Species
Alphacoronavirus (α)	Feline Infectious Peritonitis (FIP)	NC_002306.3	Cat
	Feline Coronavirus (strain UU11)	FJ938052.1	Cat
	Canine Coronavirus	KC175340.1	Dog
	Porcine Epidemic Diarrhea (PED) virus	NC_028806.1	Pig
	Transmissible Gastroenteritis (TGE) virus	NC_038861.1	Pig
	Ferret Coronavirus	NC_030292.1	Ferret
	Mink Coronavirus	NC_023760.1	Mink
	Alpaca Respiratory Coronavirus	JQ410000.1	Alpaca
Betacoronavirus (β)	Canine Respiratory Coronavirus	KX432213.1	Dog
	Bovine Coronavirus	NC_003045.1	Cattle
	White-tailed Deer Coronavirus	FJ425187.1	Whitetail Deer
	Severe Acute Respiratory Syndrome Coronavirus 2 (SARS-CoV-2)	NC_045512.2	Human
	Severe Acute Respiratory Syndrome Coronavirus (SARS-CoV)	YP_009825051.1	Human
	Middle East Respiratory Syndrome Coronavirus MERS-CoV	YP_009047204.1	Human
	MERS-like Coronavirus (PREDICT/PDF-2180)	NC_034440.1	*Pipistrelle Bat*
	Mouse Coronavirus	FJ647220.1	Mouse
	Rat Coronavirus	KF294371.1	Rat
	Rabbit Coronavirus	JN874562.1	Rabbit
	SARS-CoV-2	QLC48407.1	Tiger
	Murine Coronavirus	ACN89705.1	Mouse
	Sable Antelope Coronavirus	ABP38306.1	Sable Antelope
	Giraffe Coronavirus beta	ABP38334.1	Giraffe
	Human Coronavirus OC43 beta	AMK59677.1	Human
	Human Coronavirus OC43	AWW13551.1	Chimpanzee
	Camel Coronavirus HKU23 beta	ALA50080.1	Camel
	Buffalo Coronavirus	ANJ04717.1	Buffalo
	Equine Coronavirus	BAS18866.1	Horse
Gammacoronavirus (γ)	Avian Infectious Bronchitis	NC_001451.1	Chicken
	Turkey Coronavirus	NC_010800.1.	Turkey
	Duck Coronavirus	NC_048214.1	Duck
	Bottlenose Dolphin Coronavirus	MN690608.1	Bottlenose Dolphin
	Beluga Whale Coronavirus	NC_010646.1	Beluga Whale

**Table 2:** Host receptor and enzyme sequences included in this study

Host	Host Enzyme/ Receptor Accession Number (***Source: NCBI***)						
	ACE2	TMPRSS2	Aminopeptidase N (APN)	Sialic Acid Synthase (SAS)	Dipeptidyl Peptidase 4 (DPP4)	Furin	Cathepsin L
Human	NP_001373189.1	NP_001369649.1	NP_001368853.1	NP_061819.2	NP_001926.2	NP_001369548.1 Preproprotein	EAW62736.1
Bat	XP_032945086.1	XP_032944708.1	XP_032956109.1	XP_032979625.1	XP_011356841.1	XP_011374459.1	XP_011383262.1
Mouse	NP_001123985.1	NP_056590.2	NP_032512.2	NP_444409.1	NP_034204.1	NP_001074923.1 Preprotein	EDL16242.1
Rat	NP_001012006.1	NP_569108.2	NP_112274.1	NP_001100125.2	NP_036921.2	XP_032749231.1	BAM14518.1
Rabbit	NP_001164540.1	NP_001373057.1	NP_001075795.1	XP_002707999.1	XP_008256890.1 PREDICTED	XP_002721548.2 PREDICTED	XP_008255787.1 PREDICTED
Ferret	NP_001297119.1	NP_001373056.1	XP_012917463.1	XP_004747730.1	XP_012907449.1	XP_004763758.1	XP_012903814.1 Procathepsin L
Cat	NP_001034545.1	XP_023094479.1	NP_001009252.2	XP_023098256.1	NP_001009838.1	XP_023110662.2	XP_011286595.1 Procathepsin L
Dog	NP_001158732.1	XP_038299491.1	NP_001139506.1	XP_538746.2	XP_038302823.1	XP_038517318.1	CAC08809.1
Pig	NP_001116542.1	NP_001373060.1	NP_999442.1	NP_001172068.1	NP_999422.1	XP_020954578.1	CAC44793.1
Alpaca	XP_006212709.1	XP_031540200.1	XP_006198515.1	XP_006204124.1	XP_006196279.1	XP_031547695.1	XP_006209297.1
Cattle	NP_001019673.2	NP_001075054.1	NP_001068612.1	NP_001039947.1	NP_776464.1	NP_776561.1 Precursor	CAA62870.1
White-tailed Deer	XP_020768965.1	XP_020763597.1	XP_020765004.1	XP_020761234.1	XP_020737504.1	XP_020761451.1	XP_020760541.1
Bottlenose Dolphin	XP_019781177.2	XP_033712275.1	XP_033708027.1	XP_004322851.1	XP_033715971.1	XP_033708070.1	XP_004320974.1
Beluga whale	XP_022444817.1	XP_022408670.1	XP_022419728.1	XP_022455827.1	XP_022425590.1	XP_022419719.1	XP_030615313.1
Duck	XP_012949915.3	XP_027304175.2	XP_027322378.2	XP_027302136.1	XP_035187422.1	XP_038040754.1	XP_038026610.1 Procathepsin L
Chicken	XP_416822.2	XP_015156666.1	ACZ95799.1	NP_001007976.1	NP_001026426.2	NP_001383040.1 Precursor	NP_001161481.1 Procathepsin L
Turkey	XP_019467554.1	XP_010722229.1	XP_010715973.1	XP_010724239.2	NOT FOUND	XP_010715981.1	XP_010723392.1 Cathepsin L

## Data Availability

All data used in this study are publicly available in the National Center for Biotechnology Information (NCBI, https://www.ncbi.nlm.nih.gov/nuccore/), and the accession numbers for both virus and host sequences are provided in Tables 1 and 2.

## Abbreviations

α: Alpha
β: Beta
γ: Gamma
S: Spike
E: Envelope
M: Membrane
N: Nucleocapsid
APN: Aminopeptidase-N
ACE2: Angiotensin Converting Enzyme 2
SAS: Sialic Acid Synthase
TMPRSS2: Transmembrane Serine Protease 2
SARS-CoV-2: Acute Respiratory Syndrome Coronavirus-2
SARS-CoV: Acute Respiratory Syndrome Coronavirus
MERS-CoV: Middle East Respiratory Syndrome Coronavirus
TGE: Transmissible gastroenteritis
PED: Porcine Epidemic Diarrhea PED
FIP: Feline Infectious Peritonitis
NCBI: National Center for Biotechnology Information
